# Mixture Toxicity of Three Unconventional Gas Fracking Chemicals, Barium, *O*‐Cresol, and Sodium Chloride, to the Freshwater Shrimp *Paratya australiensis*

**DOI:** 10.1002/etc.5538

**Published:** 2023-01-13

**Authors:** Daniel J. Willems, Anupama Kumar, Dayanthi Nugegoda

**Affiliations:** ^1^ Ecotoxicology Research Group, School of Science, Bundoora West Campus Royal Melbourne Institute of Technology University Bundoora Victoria Australia; ^2^ Environment Business Unit Commonwealth Scientific and Industrial Research Organisation Urrbrae South Australia Australia

**Keywords:** Freshwater shrimp, barium, *O*‐Cresol, salinity, unconventional gas, acute toxicity

## Abstract

The 96‐h acute toxicity of barium (Ba^2+^), *o*‐cresol, and sodium chloride (NaCl) to *Paratya australiensis* was assessed in single, binary, and ternary combinations in addition to three biochemical assays: glutathione *S*‐transferase, acetylcholinesterase, and sodium–potassium adenosine triphosphatase. The 96‐h lethal concentrations that expressed 50% mortality (LC50) in the single‐toxicant exposures were Ba^2+^ = 23.4 mg/L, *o*‐cresol = 12.2 mg/L, and NaCl = 4198 mg/L. Mortality from *o*‐cresol exposure occurred between 11 and 22 mg/L, whereas Ba^2+^ was more gradual across 10–105 mg/L, and most of the NaCl mortality occurred between 2050 and 4100 mg/L. Toxic units were used to assess the binary and ternary interactions of the toxicants. A more than additive effect was observed for most combinations in the binary chemical exposures, with the ternary combinations yielding highly synergistic interactions. Greater synergism was observed with the 96‐h LC50 of *o*‐cresol in combination with the three concentrations of NaCl (1025, 2050, and 3075 mg/L) compared with Ba^2+^, with toxic units of 0.38, 0.48, and 0.10 (*o*‐cresol) and 0.71, 0.67, and 0.50 (Ba^2+^). No notable enzyme activity trends were observed in the enzyme biomarker responses from both individual and mixture exposures. Although acute single‐species toxicity tests tend to underestimate the effects of Ba^2+^, *o*‐cresol, and NaCl on populations, communities, and ecosystems in seminatural (e.g., mesocosms) and natural systems, there are currently no published acute toxicity data available for *P. australiensis* and the three toxicants used in the present study. The present study shows that chemicals with different toxicity mechanisms can potentially lead to more synergistic responses. *Environ Toxicol Chem* 2023;42:481–494. © 2022 The Authors. *Environmental Toxicology and Chemistry* published by Wiley Periodicals LLC on behalf of SETAC.

## INTRODUCTION

Unconventional gas exploration involves hydraulic fracturing, commonly referred to as *fracking*. It is used to extract natural gas from underground reservoirs. There are three classes of onshore unconventional gas reservoirs: coal bed methane, or coal seam gas in some Australian literature; shale gas; and tight gas (McGlade et al., 2013). In Australia, commercialized coal bed methane fracking practices began in 1996 in the Bowen Basin, Queensland. Currently most unconventional gas operations in Australia occur in the eastern states of Queensland and New South Wales and mostly extract natural gas from coal bed methane reservoirs. Recently, shale gas has seen development in Australia, with the first well being drilled in 2011 in the Cooper Basin, South Australia (Backé et al., 2011; Mauter et al., 2014). Reservoirs of tight gas in Australia are currently undergoing further growth and development, with few actively producing wells; most are under assessment for commercial‐scale viability (Upstream Petroleum Resources, 2016). The unconventional gas industry has seen global growth over recent decades, driven by increasing global energy demand and natural gas being a cleaner energy source than either coal or petroleum‐based energy sources as we transition to renewable energy sources.

Flowback waters and produced waters, also referred to as flowback‐produced waters (Folkerts et al., 2019), from unconventional gas exploration can contain a large variety of chemicals (Elsner & Hoelzer, 2016; National Industrial Chemicals Notification and Assessment Scheme [NICNAS], 2017; Waxman et al., 2011). Some of these chemicals are additives that form the injected hydraulic fracturing fluid, to maintain well stability and conditions. A portion of the chemicals that return in flowback‐produced water can be of geogenic origin (Apte, Kookana, et al., 2017; Apte, Williams, et al., 2017). Because there is a large variety of chemicals found in flowback‐produced water from unconventional gas extraction, a preliminary literature review was performed, resulting in three chemicals of concern to investigate further: barium (Ba^2+^; Golding et al., 2018; Jackson & Reddy, 2007; Navi et al., 2015; NICNAS, 2017; Rosenblum et al., 2017; Y. Sun, Wang, et al., 2019; Y. Sun, Yu, et al., 2019), *o*‐cresol (2‐methylphenol; Apte, Kookana, et al., 2017; Apte, Williams, et al., 2017; Bain & Kumar, 2018; Butkovskyi et al., 2017; Lester et al., 2015; Mallants et al., 2017), and sodium chloride (NaCl; Barbot et al., 2013; Benko & Drewes, 2008; Blauch et al., 2009; Estrada & Bhamidimarri, 2016; Kondash et al., 2017).

Barium is highly reactive and can be found as a variety of salts. Mineral deposits containing Ba can be exposed to injected hydraulic fracturing fluids and returned to the surface in flowback‐produced water (Rosenblum et al., 2017). It is a common drilling additive, commonly referred to as *barite* (barium sulfate, BaSO_4_; Ibrahim et al., 2017). The ion Ba^2+^ is a muscle poison at concentrations of 0.009–0.162 mg/kg Ba^2+^ as BaCl_2_ (reported as 0.0001–0.0018 mM/kg BaCl_2_) introduced into cerebrospinal fluid, resulting in tetanic spasms and convulsive seizures due to central nervous system stimulation (Ambache, 1949; Chou & Chin, 1943). In humans, an oral dose of 3.82 mg/kg Ba^2+^ as BaCl_2_ (reported as 5.8 mg/kg BaCl_2_) causes paraesthesia, muscle weakness, and flaccid paralysis (Su et al., 2020). The ion Ba^2+^ is a physiological antagonist to the potassium (K^+^) ion. Biochemically this is due to Ba^2+^ blocking K^+^ channels of the sodium–K^+^‐adenosine triphosphate ([Na^+^,K^+^]‐ATP) pump in cell membranes, ultimately causing an influx of K^+^ ions and inhibiting the passive efflux of K^+^ (McNeill & Isoardi, 2019; Oskarsson, 2015).

*O*‐Cresol (2‐methylphenol) has moderate water solubility of 2.5 g/100 ml at 25 °C (International Labour Organization, 2008), with two other isomers, *m*‐cresol (3‐methylphenol) and *p*‐cresol (4‐methylphenol). It is a geogenic contaminant of concern in unconventional gas exploration, where it is found naturally in coal tars within coal beds and can return to the surface in flowback‐produced water (Bain & Kumar, 2018). *O*‐Cresol toxicity and its associated effects include acting as a respiratory irritant, and acutely exposed animals experience neurological side effects including muscle excitation and twitching, accompanied by general fatigue (Fay et al., 2008).

High salinity is a widespread issue, where Cl^−^ and Na^+^ are typically the two most abundant ions associated with salinity in flowback‐produced water, particularly from shale gas (Liden et al., 2022; Willems et al., 2022). In Australian groundwater reservoirs associated with unconventional gas extraction, total dissolved salt concentrations range from 40 to 200 000 mg/L (Hall et al., 2018), whereas mean Na^+^ and Cl^−^ concentrations of coal bed methane waters in Australia are approximately 1300 mg/L each or total 2600 mg/L NaCl content (Willems et al., 2022). Though both Na^+^ and Cl^−^ ions are essential for life on earth, they can become toxic at sufficiently elevated concentrations with prolonged exposures or fluctuations. For freshwater organisms like *Paratya australiensis*, exposure to elevated concentrations of Na^+^ and Cl^−^ as in shale gas flowback‐produced water (Willems et al., 2022) beyond physiologically tolerable thresholds can interfere with osmoregulation. Other studies investigating salinity toxicity with *P. australiensis* have all used a synthetic marine salt with resulting 72–96‐h 50% lethal concentration (LC50) values ranging from 6600 to 39844 μS cm^−1^ (≈3693–22312 mg/L), as summarized in Paradise (2009). There are currently no published NaCl acute toxicity data for *P. australiensis*. In the present study, NaCl was used instead of synthetic marine salt (derived from marine waters) as synthetic marine salt contains SO_4_^2−^ (Atkinson & Bingman, 1997), which will react with Ba^2+^ to form the highly water‐insoluble BaSO_4_ (Golding et al., 2018). We stress that salinity toxicity to freshwater organisms is highly dependent on ionic composition (Erickson et al., 2017; Kunz et al., 2013; Mount et al., 1997, 2016, 2019), as further discussed in Cañedo‐Argüelles et al. (2013, 2016). The present study is relevant to unconventional gas waters where Na^+^ and Cl^−^ are the majority cation and anion, respectively, which is more likely in shale gas flowback‐produced waters (Willems et al., 2022).

In North America flowback‐produced waters can enter surface water environments through permitted discharges via the National Pollutant Discharge Elimination System, spills, leaks, or runoffs, as summarized in Willems et al. (2022). In Australia, which is still dominated by coal bed methane production of natural gas, 80% of flowback‐produced water from coal bed methane is beneficially reused in the agriculture industry and by reinjection into aquifers (Australian Petroleum Production and Exploration Association, 2022). Associated environmental impacts such as water management from the industry are regulated by the Environmental Protection Act 1994 (Millar et al., 2016). There have been 23 spill‐ and 10 discharge‐related incidences reported and detailed (Australian Parliament House, 2015), with spill volumes ranging from 500 to 10 000 L. We highlight that there is a paucity of published literature with an Australian context that has investigated and/or compiled data relating to these incidences and associated volumes of flowback‐produced water waste. The toxicity of the flowback‐produced water that may enter surface waters is also heavily influenced by dilutions from the surface water's volume and flow rate (McLaughlin et al., 2020; Ni et al., 2022) and further discussed (see section *Single‐chemical acute toxicity*).

Exposure of organisms to environmental contaminants may result in biochemical impairments and/or adaptive responses. Glutathione *S*‐transferase (GST) is ubiquitously distributed in all life forms and is a Phase II enzyme involved in detoxification reactions of xenobiotics in many organisms including in *P. australiensis* (Davies et al., 1994; Lekamge et al., 2019). Activity of GST is often used as a biomarker of exposure to evaluate effects caused by organic contaminants in a range of freshwater invertebrates (Dinh Van et al., 2014; McLoughlin et al., 2000; Rodrigues et al., 2015).

Acetylcholinesterase (AChE) is an enzyme found mainly at the neuromuscular junction and cholinergic synapses in the central nervous system of animals. It hydrolyzes the excitatory neurotransmitter acetylcholine into choline and acetic acid (Kim et al., 2007). Inhibition of AChE causes a buildup of acetylcholine, causing continuous and excessive stimulation of the nerves, leading to muscle spasms, paralysis, and eventual death (Fay et al., 2008; Forget et al., 2003).

Sodium–potassium adenosine triphosphatase ([Na^+^,K^+^]‐ATPase) pumps are present in all animal cells (Horisberger, 2004) and are situated in the outer plasma membrane of cells on the cytosolic side (Pivovarov et al., 2019). The (Na^+^,K^+^)‐ATPase pumps three Na^+^ out of the cell and two K^+^ into the cell for every single ATP consumed. This results in a hyperpolarization of the cell membrane, which is essential for a range of functions, as detailed in Clausen et al. (2017) and Pivovarov et al. (2019). Exposure to Ba^2+^ can cause a range of health side effects (Oskarsson, 2015), the most relevant being hypokalemic paralysis (Struyk & Cannon, 2008). Ba^2+^ is a physiological antagonist to K^+^ (Oskarsson, 2015; Tao et al., 2016). It causes a blockage of K^+^ channels of the (Na^+^,K^+^)‐ATPase pump in cellular membranes (Yellen, 1987), resulting in K^+^ transfer from extracellular to intracellular compartments. This physiologically causes decreased excitability of muscles and thus can cause hypokalemic paralysis (Oskarsson, 2015; Struyk & Cannon, 2008).

*Paratya australiensis* (Kemp, 1917), commonly known as the Australian glass shrimp, is an atyid shrimp that is located throughout southeastern Australia and survives best between 12 and 25 °C waters. It is found in freshwater systems and low‐saline estuaries and can tolerate a range of salinities, as summarized in Paradise (2009). It grazes on periphyton (Moulton et al., 2012) and is prey for fish and other large organisms (Bool et al., 2011; Ferris & Wilson, 2012; Liss et al., 2020). Because of its small size, short maturation time, and availability from the wild in southeastern Australia and through commercial suppliers, *P. australiensis* is used often to assess the toxicity of environmental contaminants in Australia, including pesticides (Abdullah et al., 1994; Davies et al., 1994; Hose & Wilson, 2005; Kumar, Correll, et al., 2010; Kumar, Doan, et al., 2010), metals (Daly et al., 1990; McDonald et al., 2020; Oulton et al., 2014; Vera et al., 2014), metal nanoparticles (Lekamge et al., 2018, 2019), and acid sulfate drainage water (Bain et al., 2016).

The primary purpose of the present study was to evaluate the 96‐h acute toxicity of single and mixture effects (binary and ternary) of the three chemicals Ba^2+^, *o*‐cresol, and NaCl found in onshore unconventional gas flowback‐produced waters to the freshwater atyid shrimp *P. australiensis*. The concentrations of Ba^2+^, *o*‐cresol, and NaCl used in the acute exposures were representative of concentrations that can be found within untreated onshore unconventional gas flowback‐produced waters. The present study compared the acute toxicity endpoints to environmentally relevant concentrations of Ba^2+^, *o*‐cresol, and NaCl found in surface waters impacted by discharges, spills, runoffs, or leaks of flowback‐produced waters. It was also of particular interest to investigate the binary or ternary toxicity involving both organic and inorganic chemicals together because few studies have investigated these combinations (Cedergreen, 2014). The shrimp were further assessed using enzyme bioassays such as GST, AChE, and (Na^+^,K^+^)‐ATPase to determine if the 96‐h exposures would result in sublethal changes in enzyme activity. We also highlight that there are no previously published acute toxicity data for *P. australiensis* with Ba^2+^, *o*‐cresol, and NaCl.

## METHODS

### Test materials

Barium as BaCl_2_ (99.999% trace metal basis, Chemical Abstracts Service [CAS] no. 10361‐37‐2), *o*‐cresol (2‐methylphenol, ReagentPlus®, ≥99%, CAS no. 95‐48‐7), NaCl (American Chemical Society [ACS] reagent, ≥99.0%, CAS no. 7647‐14‐5), and dimethyl sulfoxide (DMSO; ACS reagent, ≥99.0%, CAS no. 67‐68‐5) as a suitable low‐toxicity solvent (Organisation for Economic Co‐operation and Development [OECD], 2019) to create sufficiently concentrated stocks for *o*‐cresol were all obtained from Sigma‐Aldrich Australia. Stock solutions were prepared as follows: barium as 0.25 M of Ba^2+^ using BaCl_2_ in Milli‐Q water; *o*‐cresol as 0.5 M in DMSO (94.8% v/v DMSO) and saline (NaCl) waters were prepared to ±3% variation of nominal conductivities in dechlorinated, carbon‐filtered water (further referred to as *filtered water*) using a concentrated 5‐M NaCl stock solution (also prepared in filtered water). Nominal conductivity values were achieved using a calibrated conductivity probe (Hach HQ40d). These saline solutions were stored in new 20‐L carboys and continuously aerated. All stock solutions were made fresh the day prior to commencing each of the toxicity tests. Glassware throughout experimentation was washed in a solution of laboratory detergent (Pyroneg), rinsed, then soaked for 24 h in 5% v/v of concentrated (70%) nitric acid (HNO_3_) in Milli‐Q H_2_O, and then triple‐rinsed with Milli‐Q.

### Test organism and culture conditions

Adult *P. australiensis* were collected using a pole net with a 2‐mm mesh size along the banks of the Yarra River in North Warrandyte, Victoria, Australia (latitude 37°43′31.9454″S, longitude 145°14′8.1744″E) from March 2019 to August 2019. This is deemed a pristine site in a nature reserve area (pH 7.1 ± 0.2; dissolved oxygen >70%; temperature 16 ± 4 °C, and conductivity 225–250 μS cm^−1^). The shrimp were transported to the laboratory in plastic buckets with aeration provided. Shrimp were then divided and sorted using a dip net into clean glass aquarium tanks with 100% fresh river water with continuous aeration at a density of approximately 3 shrimp/L and brought to the set 20 ± 1 °C of the constant temperature room, set at 16: 8‐h light: dark cycle with a light intensity of 800 lux at the water surface. At 24‐h intervals for the first 72 h, partial water renewals were conducted at 50% v/v with filtered tap water and then after 96 h of acclimation a 100% renewal to filtered water. Further 25% daily renewals of filtered water were continued until 1 week of acclimation was achieved. The filtered water metal ionic composition was measured (see section *Water chemistry*). Shrimp were fed with Seramin® tropical flake food once daily (≈2% w/w) immediately after water renewals. Shrimp were not fed 24 h prior to and during the 96‐h acute toxicity test (OECD, 2019).

### Toxicity tests

Each 96‐h acute toxicity test was semistatically performed in clean 1‐L borosilicate beakers. Negative controls containing just filtered water and a DMSO solvent control when testing with *o*‐cresol were run concurrently (OECD, 2019). For each replicate, seven shrimp were used (15–23 mm in length). Test solutions were prepared to desired concentrations from stock solutions (see section *Test materials*), covered to reduce volatilization and evaporation of water, and then aerated throughout the 96‐h exposure. For single‐toxicant tests, Ba^2+^ test concentrations were a 1.8 geometric series starting from 10 mg/L for a total of five test concentrations. *O*‐Cresol test concentrations were a 2.0 geometric series starting from 1 mg/L for a total of seven test concentrations. The NaCl test concentrations were assessed at 1 025 mg/L intervals up to 6 150 mg/L (Supporting Information, Figure S1). These concentrations were determined based on range‐finding tests. During testing NaCl was converted from µS/cm to mg/L (see section *Data analysis*).

**Figure 1:**
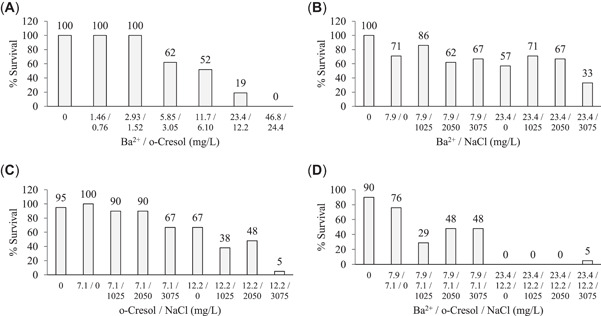
Survival at 96 h from binary and ternary toxicity tests for *Paratya australiensis*. Toxicants are separated by forward slashes. (**A**) A 2.0× geometric series from 0.0625× to 2.0× 96‐h 50% lethal concentration (LC50) values for each Ba^2+^ and *o*‐cresol (Table 1). (**B**) The 96‐h LC10 and LC50 Ba^2+^ values in combination with four selected concentrations of salinity. (**C**) The 96‐h LC10 and LC50 *o*‐cresol values in combination with four selected concentrations of salinity. (**D**) The 96‐h LC10 concentration of each Ba^2+^ and *o*‐cresol with four selected concentrations of salinity, repeated with 96‐h LC50 concentrations of each Ba^2+^ and *o*‐cresol with the three tiers of salinity. The limitations to the 96‐h LC10 and LC50 values for each Ba^2+^ and *o*‐cresol when also testing salinity were necessary to make the experiments feasible.

For binary and ternary combinations for Ba^2+^ and *o*‐cresol, LC10 and LC50 values were derived from the single (definitive) chemical tests. Sodium chloride was assessed at three selected concentrations of 1 025, 2 050, and 3 075 mg/L NaCl, which are representative of the lower range of Na^+^ and Cl^−^ in coal bed methane and shale gas waters (Willems et al., 2022). The concentrations of each of the toxicants used in the three binary tests and one ternary test and the respective 96‐h survivability rates will be outlined in (Figure 1). Additional control groups such as single‐toxicant LC10 and LC50 concentrations for Ba^2+^ and *o*‐cresol were also incorporated in these binary and ternary tests to check that mortality was consistent across the acute toxicity tests.

The four physicochemical parameters dissolved oxygen (percentage of saturation), temperature (°C), pH, and conductivity (μS cm^−1^) were measured every 24 h in all active test beakers (see section *Water chemistry*). Water samples were taken prior to water renewals for analysis of barium and *o*‐cresol concentrations. Water was 100% renewed every 24 h during the 96‐h exposure by straining of shrimp through an aquarium fish net and were out of solution <15 s, to reduce stress. After 96 h of exposure, surviving shrimp were stored at −80 °C for further biochemical analysis.

### Stability and dissolution of Ba, Na, and *o*‐cresol in test medium

Water samples were taken randomly from one replicate of each active treatment prior to renewals and were analyzed for Ba, Na, and *o*‐cresol. For Ba and Na, 9.8 ml of treatment solution was filtered into 15‐ml centrifuge tubes using 0.45‐μm syringe filters, then acidified to 2% v/v with concentrated (70%) HNO_3_. Samples that required further dilutions were diluted with 2% v/v of concentrated HNO_3_ in Milli‐Q. Metal analysis was performed using an inductively coupled plasma mass spectrometer (ICP‐MS 7700x; Agilent Technologies), a multielement standard (Agilent 5183‐4688) to determine Ba^2+^ and Na^+^ concentrations in addition to a sweep of other metals (Ag, Al, As, Be, Ca, Cd, Co, Cr, Cu, Fe, Ge, Hg, K, Mg, Mn, Mo, Ni, Pb, Rh, Sb, Sc, Se, Th, Tl, U, V, and Zn) to check water quality. Undiluted Ba and Na content was calculated for water samples, and Ba concentrations were compared with nominal test concentrations (see section *Water chemistry*).

For *o*‐cresol analysis, 50 ml of test solutions were filtered through single‐use 0.45‐μm syringe filters into acetone‐washed and three times Milli‐Q‐rinsed glass vials with no headspace and then capped with aluminum foil–lined lids. *O*‐Cresol samples were stored at 4 °C for no more than 1 week and chilled at all times during transport to the National Measurement Institute, Australia (a National Association of Testing Authorities–accredited laboratory), for analysis, which was done following the NGCMS 1111 method.

### Biochemical assays

Three biochemical assays, GST, AChE, and (Na^+^,K^+^)‐ATPase, were conducted to assess the sublethal effects of Ba^2+^, *o*‐cresol, and NaCl exposures in their single exposure, binary combinations, and the ternary mixture (see Supporting Information). For all three biochemical assays, shrimp were weighed in milligrams using an analytical balance, then homogenized (CAT; D‐79219 Staufen X 120) using 10 µL homogenizing buffer to 1 mg of shrimp mass. All three assays used 96‐well plates and a spectrophotometer (POLARstar Omega; BMG Labtech). Homogenizing buffers were prepared as described in the published literature: GST (Frasco & Guilhermino, 2002), AChE (Ellman et al., 1961), and (Na^+^,K^+^)‐ATPase (Mayer‐Gostan & Lemaire, 1991). Homogenized shrimps were then centrifuged at 4 °C (Sigma; 3‐16KL centrifuge) for 30 min at 9000*g* for GST and AChE, whereas a speed of 13 000*g* was used for (Na^+^,K^+^)‐ATPase, as described in Dogan et al. (2015), modified from Mayer‐Gostan and Lemaire (1991). The supernatants were then used to assess enzyme activity for each of the three biochemical assays. For each of the bioassays, blanks were run using the phosphate buffer required for that bioassay, substituting the sample homogenate and allowing blank corrections of data to be performed.

Activity of GST was assessed for shrimp exposed to *o*‐cresol and measured using the method described by Habig et al. (1974) adapted to a microplate by Frasco and Guilhermino (2002). For each of the supernatants from centrifugation, 100 µl was plated in each triplicate. A reaction solution containing 1‐chloro‐2,4‐dinitrobenzene and glutathione was added to wells containing supernatant just prior to plate reading. Plates were then read at 1‐min intervals for 10 min at 340 nm with path length adjusted for 300 µl. Enzyme activity was calculated as specified by Frasco and Guilhermino (2002) and expressed as micromoles of substrate hydrolyzed/min/mg protein.

Activity of AChE was assessed for shrimp exposed to *o*‐cresol and measured using the method described by Ellman et al. (1961). For each of the supernatants from centrifugation, 50 µl was plated in each triplicate in addition to 150 µl of pH 8 phosphate buffer and 25 µl of 0.01 5,5′‐dithiobis‐(2‐nitrobenzoic acid) at pH 7. Plates were then incubated at room temperature for 10 min. Finally, 25 µl of acetylthiocholine iodide was added to wells containing supernatant immediately prior to plate reading, at 412 nm every 30 s for 10 min with path length adjusted for 250 µl. Enzyme activity was calculated as specified by Ellman et al. (1961) and expressed as micromoles of substrate hydrolyzed/min/mg protein.

Activity of (Na^+^,K^+^)‐ATPase was assessed for shrimp exposed to Ba^2+^ and NaCl and measured using the method described by Mayer‐Gostan and Lemaire (1991) with modifications to centrifugation speeds as described in Dogan et al. (2015). Centrifuged supernatant (10 µl) was diluted 10‐fold in 4 °C buffered reaction and vortexed, then 10 µl of 10‐fold diluted supernatant was added to each of the triplicate wells. Dilution was necessary because the rate of reaction was too quick during incubation steps. Endpoint absorbance was read at 620 nm with path length adjusted to 210 µl.

Total protein concentration of samples for all three biochemical assays was measured according to Bradford (1976) using a 96‐well microplate. Absorbance of the spectrophotometer was set to 595 nm. A calibration curve was obtained using bovine albumin serum from which protein content of samples could be determined and then used in calculating enzyme activity on a mg protein basis.

### Data analysis

ToxRat Professional (Ver 3.0) was used to determine the lethal concentration (LC) and confidence interval values for single‐chemical exposures of Ba^2+^, *o*‐cresol, and NaCl in (Table 1). For the binary and ternary combinations of the three toxicants, the toxic unit approach was used (Marking & Dawson, 1975) with the following formula:

$$\frac{A_{m}}{A_{i}}+\frac{B_{m}}{B_{i}}=S$$

In the equation, *A* and *B* are chemicals; *i* and *m* are the toxicities (LC50, LC20, LC10) of individual chemicals and mixtures, respectively; and *S* is the sum of biological activity. This was used to assess whether the lethality of these toxicants interacting together in a system was additive (toxic unit = 1), antagonistic (toxic unit > 1), or synergistic (toxic unit < 1) relative to the lethality of the individual toxicants. One‐way analysis of variance with multiple comparison versus a control group (Holm‐Sidak method) was performed using SigmaPlot 13 to compare the biochemical activity of enzymes against control groups in each of the three biochemical assays. Statistical significance was determined at *α* = 0.05.

**Table 1:** Summary of 96‐h lethal concentration values of Ba^2+^, *o*‐cresol, and NaCl for *Paratya australiensis*

Toxicant	LC50 (95% CI)	LC20	LC10
Ba^2+^	23.4 (18.4–28.9)	11.4	7.9
*O*‐Cresol^a^	12.2 (8.0–18.4)	8.6	7.1
NaCl^b^	4198 (3681–4836)	2819	2289

^a^ Converted to the mean (68%) of the percentage of nominal as <80% of nominal achieved.

^b^ Converted from probe measured conductivity (microsiemens per centimeter) to approximately milligrams per liter from inductively coupled plasma mass spectrometric analysis.

Units are milligrams per liter.

LC*x* = *x*% lethal concentration; CI = confidence interval.

Measured electrical conductivity (μS cm^−1^) of NaCl treatments were corrected to mg/L using analytically determined (ICP‐MS) concentrations of Na^+^ to determine total added NaCl (mg/L) content, which resulted in a 0.41× factor (mean ± SE 0.41 ± 0.007) for the conversion of μS cm^−1^ into mg/L. This ensured standardized units for all three toxicants. For other literature that has cited μS cm^−1^ as measured NaCl or synthetic marine salt toxicity the conversion factor of 0.56× has been used to approximately convert μS cm^−1^ into mg/L, although this value can vary depending on various factors (Aqua‐Chem, 2019; Thermo Fisher Scientific, 2011). However, the selected factor of 0.56× best represents the majority of salinities that have been referred to in literature.

**Figure 2:**
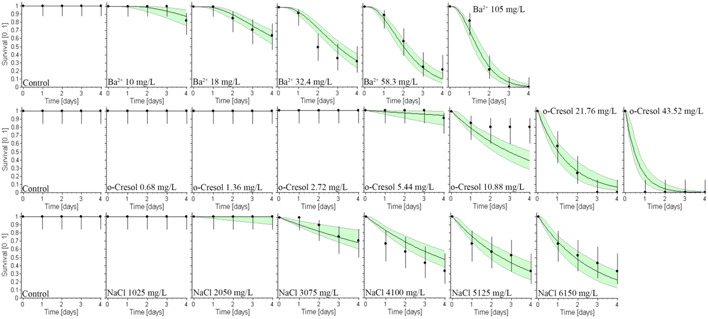
Survival at 96 h (as a fraction of the starting population) responses of *Paratya australiensis* to single‐chemical exposures of Ba^2+^ (top row), *o*‐cresol (middle row), and NaCl (bottom row) of figures. The reduced general unified threshold model for survival (GUTS)–stochastic death model set of data was used because it provided a better fit of the data. Light green areas reflect confidence intervals. The plots were generated using GUTS via the openGUTS software described in Bart et al. (2021).

The statistical software openGUTS (Ver 1.1; general unified threshold model for survival [GUTS]) was used to plot the 96‐h survival responses of *P. australiensis* to the three chemicals Ba^2+^, *o*‐cresol, and NaCl as single exposures (Figure 2). With the reduced GUTS–stochastic death model data used, we were unable to plot the binary or ternary chemical mixtures ourselves.

## RESULTS

### Water chemistry

Water quality parameters were measured daily throughout acute toxicity testing, where dissolved oxygen was >85% saturation, temperature was 18.5–19.5 °C, pH was 7.0–7.5, and conductivity of filtered water was 140 ± 20 μS cm^−1^ (≈91 ± 11.2 mg/L total dissolved salt). Where NaCl was assessed for toxicity in the tests, variability of measured conductivity of solutions was ±3% from the nominal conductivity. Across the acute toxicity tests, dissolved Ba^2+^ concentrations were >90% of nominal concentrations (Table 2), with the exception of the highest (105 mg/L Ba^2+^) concentration from the single‐chemical (definitive) test where 84% of nominal was measured. Fine white precipitate was observed at the bottom of the beakers at the 105 mg/L Ba^2+^ concentration, likely BaSO_4_. Though in further testing nominal Ba^2+^ never exceeded 46.8 mg/L (2 × 96‐h LC50 value). Reported *o*‐cresol concentrations have been reported as the average measured concentration instead of nominal (68% of nominal across 1–64 mg/L *o*‐cresol; Table 2).

**Table 2:** Nominal concentrations compared with analytically measured concentrations (mean ± SE) for Ba^2+^ and *o*‐cresol from definitive testing

Nominal concentration (mg/L)	Measured concentration (mg/L)	Nominal (%)
*Barium*		
Control	0.06 ± 0.003	NA
10	9.47 ± 0.05	95
18	16.68 ± 0.22	93
32.4	29.72 ± 0.10	92
58.3	53.58 ± 0.16	92
105	88.10 ± 1.59	84
*o*‐*Cresol*		
Control	<0.001	NA
1	0.66 ± 0.00	66
2	1.65 ± 0.05	83
4	2.00 ± 0.10	50
8	6.20 ± 0.50	78
16	11.00 ± 0.00	69
32	23.00 ± 1.00	72
64	36.50 ± 0.50	57

Definitive test concentrations are presented because these tests involved the widest range of concentrations for both Ba^2+^ and *o*‐cresol across all toxicity tests.

NA = not applicable.

The filtered water supply was also characterized for dissolved metal ions; Na = 16.9 mg/L, Mg = 6.4 mg/L, K = 2.4 mg/L, and Ca = 20.8 mg/L were the only metals that were above analytically detectable limits from the multielement standard (Agilent 5183‐4688).

### Acute survival

Survival of *P. australiensis* to single‐toxicant exposures of Ba^2+^, *o*‐cresol, and NaCl is presented in Supporting Information, Figure S1. The shrimp did not experience any mortality after 96 h in the controls. The Ba^2+^ 96‐h survivorship declined more gradually (Supporting Information, Figure S1A) across the concentrations used compared with *o*‐cresol (Supporting Information, Figure S1B). The 96‐h *o*‐cresol survivorship declined dramatically from 81% to 0% from 10.88 and 21.76 mg/L, respectively, with no mortality observed in concentrations <2.72 mg/L (Figure 2; Supporting Information, Figure S1). The highest mortality of *P. australiensis* exposed to *o*‐cresol occurs across a narrower concentration range relative to Ba^2+^. The shrimp were able to tolerate a wide range of NaCl, with 33% of shrimp being able to survive 6150 mg/L for 96 h (Supporting Information, Figure S1C). In 96‐h range‐finding, continuous exposure to NaCl at 8200 mg/L resulted in 10% of the shrimp population surviving; at higher concentrations, shrimp exposed to 12300–20500 mg/L NaCl had a distinct brown discoloration of their deceased bodies.

**Figure 3:**
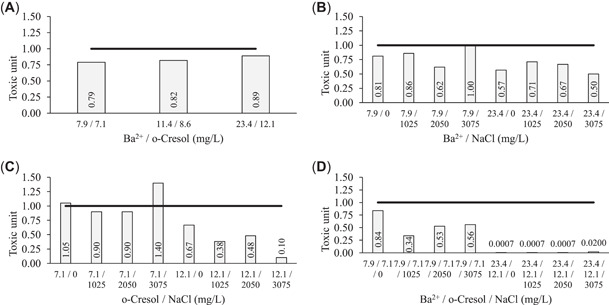
Toxic unit approach to assess the 96‐h lethality of binary and ternary combinations of Ba^2+^, *o*‐cresol, and NaCl on *Paratya australiensis*. Toxicants are separated by forward slashes and represented respectively in the horizontal axis label. The chosen salinity concentrations represent concentrations within the salinity range found in Australian coal bed methane produced water. (**A**) Interaction of Ba^2+^ and *o*‐cresol at 10%, 20%, and 50% lethal concentrations (LC10, LC20, and LC50, respectively; left to right). (**B**) Interaction of Ba^2+^ at LC10 (left four bars) and LC50 concentrations (right four bars) in combination with salinity. (**C**) Interaction of *o*‐cresol at LC10 (left four bars) and LC50 concentrations (right four bars) in combination with salinity. (**D**) Interaction of Ba^2+^ and *o*‐cresol each at LC10 (left four bars) and each at LC50 concentrations (right four bars) in combination with salinity. For (**A**), the toxic unit data were calculated against the sum of individual toxicants (Ba^2+^ and *o*‐cresol). For (**B–D**) the toxic unit data for each treatment were expressed as a ratio against the control responses (where salinity in binary and ternary mixtures was assessed). Horizontal line across each figure represents the 1 toxic unit additive threshold.

The 96‐h survival of *P. australiensis* to binary and ternary combinations of the three toxicants studied are presented in Figure 1. In water controls survivability was ≥90%. Figure 3 highlights the interaction of binary combinations and the ternary mixture of the three toxicants studied using the toxic unit approach (Marking & Dawson, 1975) and how these combinations affect the lethality of the chemicals. Shrimp exposed to a binary mixture of Ba^2+^ and *o*‐cresol had decreased survivability with increasing test concentrations (Figure 1A); however, synergistic effects (toxic unit < 1) between the two toxicants were reduced at increasing LCs (Figure 3A) relative to the lethality of the chemicals when tested individually. The two binary combinations involving NaCl (Ba^2+^ and NaCl, Figure 3B; *o*‐cresol and NaCl, Figure 3C) assess the LC10 and LC50 concentrations of each Ba^2+^ and *o*‐cresol in combination with the three selected NaCl concentrations (1025, 2050, and 3050 mg/L). The LC10 Ba^2+^ and NaCl binary test conditions showed increased synergistic effects (toxic unit = 0.62–1.00; Figure 3B) relative to the LC10 *o*‐cresol and NaCl binary test conditions (toxic unit = 0.90–1.40; Figure 3C). The opposite was observed in terms of synergistic effects at LC50 Ba^2+^ and NaCl and LC50 *o*‐cresol and NaCl conditions, where LC50 *o*‐cresol and NaCl binary combination showed greater synergistic effects (toxic unit = 0.10–0.48) compared with LC50 Ba^2+^ and NaCl binary combinations (toxic unit = 0.50–0.71). The ternary toxicity test indicates that synergism was drastically increased at 96 h even at the low LC10 concentrations of each Ba^2+^ + *o*‐cresol in each of the three concentrations of NaCl (toxic unit = 0.34–0.56; Figure 3D), whereas both Ba^2+^ and *o*‐cresol at LC50 concentrations in each of the three selected NaCl concentrations had highly synergistic effects (toxic unit = 0.0007–0.02).

### Biochemical assays

In 96‐h single exposures to Ba^2+^, *o*‐cresol, and NaCl, GST, AChE, and (Na^+^,K^+^)‐ATPase were evaluated in surviving *P. australiensis*, as presented in Supporting Information, Figure S2. Binary and ternary chemical exposures were also assessed with the same biochemical assays but not included because there were no remarkable results (see Supporting Information). Exposures involving *o*‐cresol were assessed using GST and AChE. Exposures involving Ba^2+^ and NaCl were assessed with (Na^+^,K^+^)‐ATPase. Replication was *n* = 3 for the majority of treatments, except where limited 96‐h survivors were available (*n* = 2).

Activity of GST (Supporting Information, Figure S2A) was hypothesized to increase in shrimp with increased exposure concentrations of *o*‐cresol relative to the control group. However, this was not observed; despite activity having increased relative to the controls in each of the exposure concentrations, the increases in GST activity fluctuated between test concentrations. Some significant differences were observed (*α* < 0.05) at 0.68 and 1.36 mg/L *o*‐cresol relative to the control group, though 2.72 mg/L exposure showed no significant difference in activity despite having higher mean activity compared with the 1.36 mg/L exposure. This was attributed to the GST activity within each shrimp at a given test concentration being highly variable with insufficient statistical power and insufficient replication (*n* = 3). No significant differences in AChE activity (Supporting Information, Figure S2B) compared with the control group were observed in shrimp exposed to *o*‐cresol. There were no significant differences in (Na^+^,K^+^)‐ATPase activity (Supporting Information, Figure S2C) relative to the control group in each of the Ba^2+^ test concentrations tested. It was hypothesized that (Na^+^,K^+^)‐ATPase activity would decrease with increased Ba^2+^ exposure concentrations relative to the controls given that Ba^2+^ is a known inhibitor of the (Na^+^,K^+^)‐ATPase pump. No significant differences were observed for (Na^+^,K^+^)‐ATPase activity (Supporting Information, Figure S2D) for shrimp exposed to NaCl relative to the control group. The replication of *n* = 3 for biochemical analysis of *P. australiensis* was performed because of 96‐h shrimp having to be split across multiple biomarkers.

## DISCUSSION

### Single‐chemical acute toxicity

There are currently no Australian freshwater default guideline values for Ba, *o*‐cresol, and NaCl (Australian and New Zealand Governments [ANZG], 2018). For Ba in freshwaters in The Netherlands, it has been proposed to set (long‐term) annual average environmental quality standards (EQS) to 93 µg/L, while in the short term the maximum acceptable concentration EQS (MAC‐EQS) is 1.1 mg/L (Verbruggen et al., 2020). Though there are no default guideline values for *o*‐cresol, there are for the chemically similar phenol (ANZG, 2018). The lowest and highest protection thresholds for phenol at 80% and 99% are, respectively, set at 1.2 and 0.085 mg/L, though cresols are typically more toxic than phenol (Duan et al., 2018; Zhou & Nemati, 2018). For NaCl, there are North American (US and Canadian) acute chloride guidelines for freshwater biota of 860 mg Cl^−^/L (US Environmental Protection Agency [USEPA], 1988) and 640 mg Cl^−^/L (Canadian Council of Ministers of the Environment [CCME]), 2011).

The proposed Ba MAC‐EQS value of 1.1 mg/L is approximately sevenfold lower than the 96‐h Ba^2+^ LC10 value of 7.9 mg/L for *P. australiensis* (Table 1), which would provide high levels of protection for *P. australiensis* and other similarly Ba^2+^‐tolerant species (Supporting Information, Table S1). Sensitive species such as the New Zealand mud snail (*Potamopyrgus jenkinsi*) with a Ba^2+^ 96‐h LC50 of 0.33–1.74 mg/L could still be at elevated risk of increased acute mortality.

Using the chemically similar and least protective (80%) phenol guidelines (ANZG, 2018) for *o*‐cresol, 1.2 mg/L would provide a high level of protection for *P. australiensis* because its 96‐h LC10 of 7.1 mg/L is approximately sixfold higher. The lowest observable effect concentration for *P. australiensis* mortality was between 2.72 and 5.44 mg/L (Figure 2). Other crustaceans (Supporting Information, Table S2) should be similarly protected with comparable LC50 values to *P. australiensis* in the present study.

A comparison can be made between NaCl (mg/L) toxicity data in the present study and the mg Cl^−^/L guideline values. The NaCl 96‐h LC10 of 2 289 mg/L would be equivalent to 1 387 mg Cl^−^/L (assuming Cl makes up 60.6% of the mass of NaCl), which exceeds the lower guidelines values of 860 mg Cl^−^/L (USEPA, 1988) and 640 mg Cl^−^/L (CCME, 2011). Though *P. australiensis* and many other freshwater organisms can tolerate a wide range of salinities (Paradise, 2009) and will exceed the guideline values, small proportions of other major ions will influence the toxicity of NaCl (Mount et al., 2016).

The concentrations of Ba, *o*‐cresol, Na, and Cl in unconventional gas and oil produced waters occur across a wide range, with 13 800 mg Ba/L (Barbot et al., 2013), *o*‐cresol at 554.6 mg/L (Sun et al., 2013), 150 000 and 250 000 mg/L Na^+^ and Cl^−^, respectively (Benko & Drewes, 2008). Though these high concentrations found in produced waters will be diluted if discharged or spilled into surface waters, dilution is greatly influenced by volumes and flow rates of the surface water body and the flowback‐produced water. Toxicity will be more prominent closer to the point of discharge or where the spill meets the surface water body.

Barium has been measured in proximal surface waters impacted by unconventional oil and gas discharges or spills (Bonetti et al., 2021; Lauer et al., 2016; McLaughlin et al., 2020; Ni et al., 2022; Warner et al., 2013). Warner et al. (2013) found mean Ba concentrations at 13.4 mg/L at the point of effluent from a brine‐treatment plant, with a decline to 10.9 mg/L at 1–20 m downstream; and at 20–300 m downstream concentrations had sharply declined to 0.93 mg/L. McLaughlin et al. (2020) found Ba concentrations in treated produced water discharge at approximately 0.14 mg/L at the discharge point; 15 km downstream they had halved to approximately 0.07 mg/L, and once the discharge stream had entered the perennial river (∼2–5 km from the intersection of two water bodies), concentrations had further dropped to 0.04 mg/L, which was similar to concentrations found at a control site. Lauer et al. (2016) collected 29 surface water samples (divided into two classes, Types A and B spills) from the Bakken region in North Dakota that were either known or suspected to have been impacted by unconventional oil and gas extraction. They found dissolved Ba concentrations at approximately 0.17 and 0.2 mg/L, Types A and B, respectively, whereas produced water associated with these spills had a Ba content of 13.5 mg/L. The sharp decrease in dissolved Ba concentrations from the produced waters to the surface waters is likely due to the high dissolved SO_4_ concentrations (1 000 and 3 000 mg/L, Types A and B, respectively) present in uncontaminated background waters of the region, which will react with dissolved Ba to form highly insoluble BaSO_4_ precipitate.

The Ba concentrations in surface waters impacted by discharges and spills of unconventional oil and gas flowback‐produced water are at concentrations similar to or lower than the LC10 and LC20 of 7.9 and 11.4 mg/L, respectively, for *P. australiensis* (Table 1), though these concentrations could have much greater risk to more Ba‐sensitive species (*Potamopyrgus jenkinsi*; Supporting Information, Table S2). We also stress that in an environment where water chemistry is complex, other major and trace ions will influence the toxicity of Ba. Untreated flowback‐produced waters, particularly from spills or leaks during transport, pose a great risk (Vengosh et al., 2014). Particular flowback‐produced waters are likely to have greater Ba toxicity risk. These include more saline waters (i.e., shale gas), which typically correspond with higher Ba concentrations (Willems et al., 2022), in combination with low SO_4_ concentrations in both flowback‐produced water and surface waters, which would reduce BaSO_4_ precipitation. The freshwater alga *Chlorella* sp. 12 was shown to be significantly more sensitive to precipitated Ba (48‐h LC10 3.5 mg/L) than dissolved Ba (48‐h LC10 40 mg/L), thus possibly impacting food webs, with Ba concentrations found in flowback‐produced waters (Golding et al., 2018; Willems et al., 2022) and even in surface waters more directly impacted by unconventional oil and gas discharge or spills of flowback‐produced waters.

There is a paucity of data relating to *o*‐cresol in surface waters impacted by unconventional oil and gas, with McLaughlin et al. (2020) being the sole study. Concentrations were found at 0.00236 and 0.00241 mg/L across two different sampling periods at the point of discharge and persisted 1.4 km in the discharge stream. Fay et al. (2008) state that cresols in general are typically at ≤0.001 mg/L when detected in surface waters, and biodegradation is the dominant mechanism for rapid breakdown of cresol in waters and thus will not persist in the environment. The approximate 0.0024 mg/L concentrations found in McLaughlin et al. (2020) are approximately 3000‐fold less than the *o*‐cresol 96‐h LC10 of 7.1 mg/L for *P. australiensis* in the present study, with most other freshwater invertebrates acutely exposed to *o*‐cresol generally being more tolerant (Supporting Information, Table S2).

The ions Na^+^ and Cl^–^ that form NaCl can be the dominant ions associated with flowback‐produced waters from unconventional gas, in particular shale gas (Willems et al., 2022). Discharge or spills of these waters into surface waters will impact surface water salinity or, more specifically for the present study, the Na and Cl concentrations of these waters. Several studies have investigated Na and Cl concentrations in surface waters impacted by unconventional oil and gas discharges or spills (Lauer et al., 2016; McLaughlin et al., 2020; Ni et al., 2022; Warner et al., 2013). The study by Lauer et al. (2016) found mean Na and Cl concentrations in flowback‐produced water at 38 700 and 88 500 mg/L, respectively, whereas in proximal spill sites (by two classes/types of spills) on average waters contained Na at 3720 and 1840 mg/L, and Cl was 7040 and 5040 mg/L, resulting in Na being diluted by approximately 10‐ to 20‐fold and Cl by 12‐ to 18‐fold from flowback‐produced water to surface waters impacted by the spills. Warner et al. (2013) investigated discharges from an shale gas flowback‐produced water brine‐treatment plant. At effluent point of release, Na was 27300 mg/L and declined to 1750 mg/L (15‐fold dilution) at 1–20 m downstream and by >300 m downstream had reached background levels of 21 mg/L, whereas Cl saw a less pronounced decline from 80 500 mg/L at effluent source to 16 200 mg/L (five fold dilution) at 1–20 m downstream, though this was only slightly elevated relative to a background site at >300 m downstream. Similar spatial trends were observed in Ni et al. (2022). Though McLaughlin et al. (2020) observed a slight increase of Na and Cl 15 km downstream from a produced water treatment facility's effluent, Na and Cl were both elevated relative to the background concentrations at 15‐ to 30‐fold and 30‐ to 40‐fold, respectively. We stress that Na and Cl toxicity are both influenced by concentrations of other major ions (Erickson et al., 2017; Kunz et al., 2013; Mount et al., 1997, 2016, 2019), as discussed in depth in Cañedo‐Argüelles et al. (2013, 2016) at concentrations found in both flowback‐produced water and surface waters. This is particularly important with *P. australiensis* and its sensitivity to NaHCO_3_, where it is 35‐ to 50‐fold more sensitive to NaHCO_3_ compared with synthetic marine salt (Hills et al., 2019). Also, coal bed methane flowback‐produced waters have elevated HCO_3_^−^ concentrations (Willems et al., 2022) and if discharged into surface waters, *P. australiensis* could be at increased risk.

Multiple studies have investigated acute salinity toxicity with *P. australiensis* using synthetic marine salt, as summarized in Paradise (2009), though none have used NaCl as in the present study. The synthetic marine salt acute toxicity data for *P. australiensis* ranges from 6 600 μS cm^−1^ or approximately 3700 mg/L (Bacher & Garnham, 1992) to a maximum of 39844 μS cm^−1^ or approximately 22 300 mg/L (Paradise, 2009). In the present study the NaCl 96‐h LC50 value of 4 198 mg/L (Table 1) is loosely comparable to the synthetic marine salt 96‐h LC50 of approximately 3 700 mg/L (Bacher & Garnham, 1992). *Paratya australiensis* used in Bacher and Garnham (1992) were also collected from the Yarra River, as done in the present study. The wide range of synthetic marine salt 72‐h and 96‐h LC50 values for *P. australiensis* could be attributed to environmental and climate factors (Nielsen et al., 2003). In addition, geographical separation and isolation of *P. australiensis* populations likely result in a complex of cryptic species of *P. australiensis* (Cook et al., 2006).

### Binary and ternary chemical acute toxicity

An organism in the environment is rarely exposed to a single chemical; typically, exposure is to many chemicals. Binary and ternary toxicity of chemical mixtures is used to investigate these interactions in freshwater invertebrates in various classes of chemicals, including metals (Cooper et al., 2009; McDonald et al., 2020; Rosenfeldt et al., 2016) and pesticides (Arora & Kumar, 2015; Pham et al., 2018). In the present study it was of interest to determine if there were any additive, synergistic, or antagonistic interactions between the inorganic chemicals (Ba^2+^ and NaCl) and organic (*o*‐cresol) in their binary combinations and the ternary mixture.

In the Ba^2+^ and *o*‐cresol binary exposure (Figure 3A), the interaction of the two chemicals was slightly synergistic at the LC10 concentrations (toxic unit = 0.79) and became slightly less synergistic at the LC50 concentrations (toxic unit = 0.89). The results confirm the hypothesis that the combination of these chemicals would likely be synergistic because the two chemicals are dissimilar.

In the Ba^2+^ and NaCl binary exposure (Figure 3B), greater synergism occurred between the two toxicants at the 96‐h LC50 Ba^2+^ combinations than in the 96‐h LC10 Ba^2+^ combinations. This response was expected given the increased concentrations of Ba^2+^, whereas concentrations of NaCl were maintained the same across 96‐h LC10 and 96‐h LC50 combinations. Like the Ba^2+^ and NaCl binary test, the *o*‐cresol and NaCl binary test (Figure 3C) shows greater synergism occurring at 96‐h LC50 *o*‐cresol and NaCl combinations compared with the 96‐h LC10 *o*‐cresol and NaCl.

It is important to highlight the greater synergistic response (toxic unit = 0.10–0.48) across the *o*‐cresol 96‐h LC50 and NaCl combinations compared with the Ba^2+^ 96‐h LC50 and NaCl combinations (toxic unit = 0.5–0.71), where NaCl concentrations were the same across the two tests. The differences in responses between these two binary tests could be attributed to the presence of Na^+^ in solution that may partially inhibit the binding of Ba^2+^ with the potassium inward rectifier channels in the (Na^+^,K^+^)‐ATPase pumps (Bhoelan et al., 2014), which is the primary mode of action of Ba^2+^ toxicity. The elevated concentrations of Na^+^ in solution may make conditions more favorable for the (Na^+^,K^+^)‐ATPase pumps in cells of *P. australiensis* to maintain the normal cellular biochemistry of high Na^+^ outside and low inside cells for the (Na^+^,K^+^)‐ATPase pump to function properly (Pivovarov et al., 2019), thus reducing the negative effects of Ba exposure. Because *o*‐cresol and Na^+^ are chemically dissimilar, they do not have this interaction, thus the greater synergistic response in the 96‐h LC50 *o*‐cresol and NaCl exposures compared with the 96‐h LC50 Ba^2+^ and NaCl test exposures. The review by Heugens et al. (2001) also states that metal toxicity is generally negatively correlated with salinity (i.e., higher salinity generally increased metal toxicity), whereas no clear trend was observed for organic chemicals (except for organophosphate insecticides).

In the ternary test (Figure 3D) at 96‐h LC10 concentrations for Ba^2+^ and *o*‐cresol without additional NaCl the lowest synergistic response in this test was observed (toxic unit = 0.84). With the addition of NaCl at the three concentrations (1025, 2050, and 3075 mg/L) in combination with 96‐h LC10 concentrations of each Ba^2+^ and *o*‐cresol, synergism was greatly increased (toxic unit = 0.34–0.56). This indicates that when shrimp are stressed by exposure to Ba^2+^ and *o*‐cresol additional NaCl can compound the toxicity of Ba^2+^ and *o*‐cresol, considering the shrimp had 100% survivorship after 96‐h exposure at 2 050 mg/L NaCl (Supporting Information, Figure S1). Synergism was even more pronounced at the 96‐h LC50 Ba^2+^ and 96‐h LC50 *o*‐cresol in combination with the same three concentrations of NaCl, with an extremely synergistic response observed (toxic unit = 0.0007–0.02). There is a paucity of data on binary toxicity of metals and organics (e.g., in the present study, Ba–metal, *o*‐cresol–organic). Most binary or ternary toxicity literature refers to multiple chemicals in a single class/group (metal–metal, pesticide–pesticide, etc.), making comparisons between species difficult.

### Sublethal toxicity of Ba, *o*‐cresol, and NaCl

Overall the biochemical assays were mostly inconclusive and did not yield significant results or clear trends (Supporting Information, Figure S2). Several factors likely contributed to this. The *P. australiensis* in the present study were collected from the field and were quite variable in size and hence possibly age and sex. This was mitigated as best as possible by trying to select shrimp of similar sizes while in the field and avoid collecting gravid individuals. Given that a wild population was used, it would be reasonable to expect greater genetic diversity, and thus varying responses, resulting in high variability of enzyme activity in individuals. The *n* = 3 replication was insufficient, despite prior studies using the same replication for biochemical assays (Lekamge et al., 2018, 2019). Greater replication for the biochemical testing would greatly improve statistical power and enable potential outliers to be identified and removed if necessary; thus, a better evaluation of biochemical responses could then be used to form more conclusive comparisons to other related work.

## CONCLUSION

Concentrations of Ba, *o*‐cresol, Na, and Cl in surface waters impacted by unconventional oil and gas are usually greatly diluted or precipitated (e.g., Ba) by the surface water body. Concentration gradients exist from effluent or spill origin (high) to farther downstream (low), reducing the toxicity of dissolved or suspended chemicals farther away from the origin. Other literature has shown that the highly water‐insoluble BaSO_4_ has greatly increased toxicity to the alga *Chlorella* sp. 12 species compared with dissolved Ba, thus potentially reducing the abundance of primary producers in freshwater environments impacted by BaSO_4_ precipitation and consequently impacting organisms higher in these food webs. Despite water‐treatment options being available for flowback‐produced waters, they have not always shown complete removal of Ba before the water is discharged; thus, Ba is still a risk to surface waters. *O*‐Cresol concentrations in surface waters impacted by unconventional oil pose a low risk because of rapid biodegradation. High‐salinity untreated flowback‐produced waters that enter surface waters from spills pose an increased risk, particularly to freshwater organisms with greater sensitivity to NaCl or total salinity. Treated flowback‐produced waters discharged to surface waters might still be sufficiently saline to impact freshwater biota close to the effluent discharge point but risk decreases with distance downstream because of dilution. We also highlight that the complex water chemistry of surface waters and flowback‐produced waters will likely influence the toxicity of Ba, *o*‐cresol, and NaCl. Consequently, single‐species laboratory exposures likely underestimate the effects of these chemicals on populations, communities, and ecosystems.

Further research is needed to better understand *o*‐cresol toxicity to freshwater invertebrates and its presence in flowback‐produced waters. In the future when performing enzyme bioassays it is crucial to ensure that sufficient replication is used, such as by using a more refined exposure regime (i.e., fewer treatments or lower exposure concentrations) but increased replication for each of these treatments to ensure enough 96‐h survivors for sufficient replication for the bioassays and resulting sufficient statistical power to determine possible changes in enzyme activity. We saw no remarkable trends due to great variability in responses in *P. australiensis* individuals with limited replication (*n* = 3).

## Supporting Information

The Supporting Information is available on the Wiley Online Library at https://10.1002/etc.5538.

## Disclaimer

The authors declare no competing financial interest.

## Author Contributions Statement

**Daniel J. Willems**: Conceptualization; Data curation; Formal analysis; Investigation; Methodology; Project administration; Visualization; Writing—original draft; Writing—review & editing. **Anupama Kumar**: Conceptualization Data curation; Formal analysis; Supervision; Visualization; Writing—review & editing. **Dayanthi Nugegoda**: Conceptualization; Supervision; Writing—review & editing.

## Supporting information

This article includes online‐only Supporting Information.

Supporting Information (Supplemental Tables Draft ETCJ‐Aug‐22‐00498.R2).

Supporting Information (Supplemental) Figures (Draft ETCJ‐Aug‐22‐00498.R2).

## Data Availability

Additional data are available online at https://doi.org/10.17632/s62cyycbd3.1.
