# Supplementary material for: Mixture Toxicity of Three Unconventional Gas Fracking Chemicals, Barium, O‐Cresol, and Sodium Chloride, to the Freshwater Shrimp Paratya australiensis
Source: Environ Toxicol Chem. 2023 Jan 13;42(2):481–94. doi: 10.1002/etc.5538 (PMC10107621; doi:10.1002/etc.5538)
Supplement: Supplementary file 2 — Supporting Information (Supplemental) Figures (Draft ETCJ‐Aug‐22‐00498.R2). [file ETC-42-481-s002.docx]

**Figure S1**: 96-h survival plot at 24h intervals of *Paratya australiensis* for single chemical exposures of A) Ba^2+^, B) o-Cresol and C) NaCl. All units mg/L. Note that there is data overlap for lower concentration treatments in o-Cresol and NaCl.

**Figure S2**: Changes in enzyme activity as % activity relative to the H_2_O control groups for 96-h exposed *P. australiensis* to Ba^2+^, o-Cresol and NaCl as single toxicant exposures. (A) GST activity shrimp exposed to o-Cresol, (B) AChE activity shrimp exposed to o-Cresol, (C) (Na+, K+)-ATPase activity shrimp exposed to Ba^2+^, (D) (Na+, K+)-ATPase activity shrimp exposed to NaCl. (α < 0.05, n = 3). Letter sign denotes significance between the test concentration and control group. All concentrations are in mg/L
